# Does the type of surgical approach affect the clinical outcome of total knee arthroplasty?

**DOI:** 10.1007/s00132-021-04068-x

**Published:** 2021-02-11

**Authors:** Ricarda Lechner, Matteo Lazzeri, Wilhelm Oberaigner, Paul Nardelli, Tobias Roth, Paul Köglberger, Martin Krismer, Michael Christian Liebensteiner

**Affiliations:** 1 Department of Orthopaedics and Traumatology, Medical University Innsbruck, Innsbruck, Austria; 2 Institut für klinische Epidemiologie, Tirol Kliniken, Innsbruck, Austria; 3 UMIT—Private University for Health Sciences, Medical Informatics and Technology, Hall, Austria; 4 Department of Anesthesiology and Critical Care Medicine, Klinikum Wels—Grieskirchen, Wels, Austria; 5 Division of Intensive Care and Emergency Medicine, Department of Internal Medicine, Medical University Innsbruck, Innsbruck, Austria

**Keywords:** Prosthesis survival, Surgical procedures, operative, Knee surgery, Range of motion, Functional outcome, Prothesenüberleben, Operationsverfahren, Kniechirurgie, Bewegungsumfang, Funktioneller Outcome

## Abstract

**Background:**

The aim of the study was to investigate the issue of medial midvastus (MMV) vs. medial parapatellar (MPP) approaches in total knee arthroplasty (TKA). It was hypothesized that the two surgical approaches would produce significantly different results with respect to patient-reported knee score outcome (hypothesis 1), short-term postoperative range of motion (ROM) (hypothesis 2), long-term postoperative ROM (hypothesis 3) and prosthesis survival (hypothesis 4).

**Methods:**

A retrospective comparative study design was applied. Data sets were obtained from the state arthroplasty registry. The Western Ontario and McMaster Universities osteoarthritis index (WOMAC) data were analyzed from preoperative and 1 year postoperatively. The ROM data were analyzed for the time points preoperative, postoperative days 4 and 10 and 1 year.

**Results:**

Available were 627 cases (407 MMV vs. 220 MPP) and 1 year postoperatively there were no significant differences between groups regarding the WOMAC scores (hypothesis 1). Early postoperatively on days 4 and 10 after TKA there were no differences between groups (*p* = 0.305 and *p* = 0.383, respectively, hypothesis 2). Likewise, ROM did not significantly differ between the groups 1 year after TKA (*p* = 0.338, hypothesis 3). The 5‑year prosthesis survival did not differ between the groups and showed 94.46% (95% confidence interval, CI 90.69–96.73%) in the MMV group and 94.33% (95% CI 89.96–96.83%) in the MPP group (*p* = 0.664, hypothesis 4).

**Conclusion:**

Both surgical approaches produce equivalent clinical results in terms of early postoperative ROM, late postoperative ROM and 1‑year WOMAC. The same prosthesis survival rates can be expected.

## Abstract (de)

**Hintergrund:**

Ziel der Studie war es, den medialen Midvastus- (MMV) vs. medialen parapatellaren (MPP) Zugang bei der Knietotalendoprothetik (TKA) zu untersuchen. Es wurde die Hypothese aufgestellt, dass die beiden operativen Zugänge zu signifikant unterschiedlichen Ergebnissen hinsichtlich des vom Patienten berichteten Kniescores (Hypothese 1), des kurzfristigen postoperativen Bewegungsumfangs (ROM; Hypothese 2), des langfristigen postoperativen ROM (Hypothese 3) und des Prothesenüberlebens (Hypothese 4) führen würden.

**Methoden:**

Ein retrospektiv vergleichendes Studiendesign wurde angewandt. Die Datensätze wurden aus dem staatlichen Endoprothesenregister entnommen. Die Daten des Western Ontario and McMaster Universities Osteoarthritis Index (WOMAC) wurden präoperativ und 1 Jahr postoperativ analysiert. Die ROM-Daten wurden für die Zeitpunkte präoperativ, postoperativ Tag 4 und 10 sowie 1 Jahr analysiert.

**Ergebnisse:**

Es standen 627 Fälle zur Verfügung (407 MMV vs. 220 MPP), und 1 Jahr postoperativ gab es keine signifikanten Unterschiede zwischen den Gruppen hinsichtlich der WOMAC-Scores (Hypothese 1). Früh postoperativ an Tag 4 und 10 nach TKA gab es keine Unterschiede zwischen den Gruppen (*p* = 0,305 bzw. *p* = 0,383; Hypothese 2). Ebenso unterschied sich das ROM 1 Jahr nach TKA nicht signifikant zwischen den Gruppen (*p* = 0,338; Hypothese 3). Das 5‑Jahres-Prothesenüberleben unterschied sich nicht zwischen den Gruppen und zeigte 94,46% (95% Konfidenzintervall [KI] 90,69–96,73%) in der MMV-Gruppe und 94,33% (95% KI 89,96–96,83%) in der MPP-Gruppe (*p* = 0,664; Hypothese 4).

**Schlussfolgerung:**

Beide chirurgischen Ansätze führen zu gleichwertigen klinischen Ergebnissen in Bezug auf frühes postoperatives ROM, spätes postoperatives ROM und 1‑Jahres-WOMAC. Es können die gleichen Prothesenüberlebensraten erwartet werden.

## Introduction

Up to 30% of patients were reported to dissatisfied with the outcome of total knee arthroplasty (TKA) because of unexplained pain [9, 15]. When discussing patient dissatisfaction following TKA a differentiation can be made between implant-related, patient-related and surgery-related factors [8, 12, 22].

Regarding surgery-related factors previous researchers discussed the role of different surgical approaches on outcome after TKA [11, 13, 18]. So far, 12 studies have compared the medial midvastus approach (MMV) and the medial parapatellar approach (MPP) and provided several clinical outcome parameters after TKA [1, 2, 4–6, 10, 14, 17, 19, 20, 23, 25]. Those studies were in part incongruent in that six of those studies favoured the MMV approach [1, 2, 4, 19, 23, 25], while the other six studies reported no such advantages [5, 6, 10, 14, 17, 20]. There seems to be a slight advantage of the MMV approach in terms of quadriceps strength, active straight leg raise and partially also patient-reported outcomes only in the very early postoperative period (weeks 1 and 2). With respect to sample size most of those publications provided 20 or 30 patients per group.

Due to the abovementioned conflicting evidence and the rather small sample sizes it was the aim of the study to investigate the issue of MMV vs. MPP approach in TKA once again but on the basis of much larger study populations. It was hypothesized that the two surgical approaches would lead to significantly different results with respect to patient-reported knee score outcome (hypothesis 1), short-term postoperative ROM (hypothesis 2), long-term postoperative ROM (hypothesis 3) and prosthesis survival (hypothesis 4).

## Material and methods

**Fig. 1:**
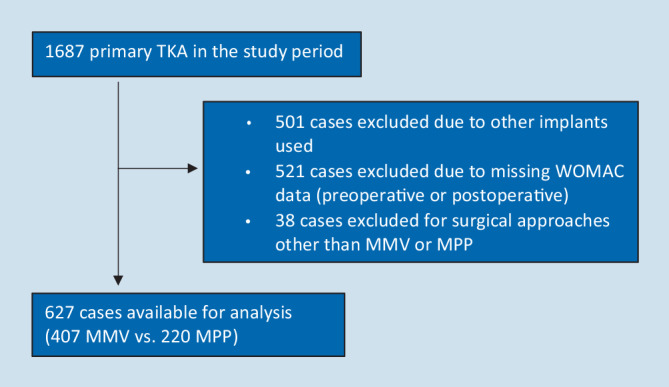
Flow chart of excluded patients. *TKA* total knee arthroplasty, *MMV* medial midvastus approach, *MPP* medial parapatellar approach, *WOMAC* Western Ontario and McMaster Universities osteoarthritis index

A retrospective comparative study design was applied. Data available from clinical routine were analyzed after approval by the IRB of the authors’ affiliated institutions. Patients who previously underwent primary TKA as part of the clinical routine were analyzed. Data sets were obtained from the state arthroplasty registry and covered all primary TKA cases performed at Department of Orthopaedics and Traumatology, Medical University of Innsbruck, Innsbruck, Austria from 2008 to 2015. Cases were excluded in the case of a) surgical approaches other than MPP and MMV, b) implants other than Scorpio CR or Triathlon CR (Stryker, Kalamazoo, MI, USA), c) incomplete preoperative WOMAC data and d) incomplete postoperative WOMAC data (1 year) (Fig. 1).

Information on whether an MPP or an MMV approach had been applied was extracted from the arthroplasty registry database. While one group of surgeons routinely performed the MMV approach another group routinely used the MPP approach for primary TKA. The prosthesis was implanted according to the manufacturer’s instructions using a measured resection technique with standard cutting blocks and instruments. Intramedullary referencing was applied at the femur and extramedullary referencing at the tibia. In accordance with the hospital’s clinical routine, the patella was left unresurfaced. All operations were performed by consultant orthopedic surgeons specialized in knee arthroplasty or under the supervision of one of these surgeons. Patient positioning, antibiotic and deep vein thrombosis prophylaxis, draping, and tourniquet control were standardized. All patients underwent the same standardized rehabilitation program after surgery. Patients were mobilized from the first postoperative day under supervision of the physiotherapists of the university hospital. Exercises included continuous passive motion, assisted and unassisted knee extension, walking and stair climbing with two crutches and progression as tolerated. For patient-reported outcome measurement the Western Ontario and McMaster Universities osteoarthritis index (WOMAC score) [3] was available from the arthroplasty registry. The questionnaire was applied in the German language version [24] (main outcome parameter). The questionnaire was completed on the day before surgery and postoperatively 1 year after surgery. The WOMAC questionnaire collected data on pain, stiffness, and physical function. Every item was completed on an 11-point scale and converted for analysis purposes to a scale from 0 to 100, 0 denoting the best and 100 the worst response. The score for each of the three main dimensions was defined as the sum of all item scores divided by the number of items. The total score was defined as the sum of pain, stiffness and function scores divided by three.

The ROM data collected with goniometers during clinical routine were taken from the medical records for the following points in time: preoperative, postoperative days 4 and 10 and 1 year.

As descriptive measures for WOMAC scores and ROM at different time points we estimated mean value and standard deviation. Differences between groups were tested for preoperative and postoperative WOMAC scores applying the Mann-Whitney U-test and for ROM applying the t‑test. Prosthesis survival was estimated with the Kaplan-Meier method from date of surgery until date of revision, data of death or end of follow-up (31 December 2015), whichever occurred first. We report Kaplan-Meier estimates for prosthesis survival at 1–5 years together with 95% confidence intervals (CI). Differences in survival curves were tested using the log-rank test. Data analysis was performed with Stata Version 13 (StataCorp LP, College Station, TX, USA). Alpha was defined as 0.05 (two-tailed tests).

## Results

Available for analysis were 627 cases (407 MMV vs. 220 MPP). In the MMV group the mean age was 70.1 ± 9.1 years, 64.1% of the participants were female and in 43.7% the left side was operated. In the MPP group the mean age was 69.0 ± 9.7 years, 57.7% of the participants were female and in 45.5% the left side was operated. None of these demographic parameters differed significantly between the groups. Preoperative leg axis (mFTA) from whole leg radiographs was also similar between the groups (Table 1).

**Table 1:** Mechanical femorotibial angle (mFTA) and WOMAC score and preoperative range of motion (ROM), short-term postoperative ROM (days 4 and 10), WOMAC score and long-term postoperative ROM (1 year) for both groups

	MMV		MPP		
	Mean	SD	Mean	SD	*p*-value
mFTA preop	182.6	6.6	182.2	7.1	0.479
WOMAC pain preop	48.7	21.0	49.7	20.8	0.3704
WOMAC stiffness preop	54.4	25.9	52.3	25.4	0.3128
WOMAC function preop	51.5	20.8	51.5	21.2	0.9060
WOMAC total preop	51.6	20.0	51.2	19.7	0.9216
ROM preop (°)	109.5	16.2	108.0	15.6	0.282
ROM day 4 (°)	66.8	14.6	68.1	15.2	0.305
ROM day 10 (°)	88.6	11.8	87.5	12.5	0.383
WOMAC pain 1 year	15.7	18.9	17.2	20.1	0.6952
WOMAC stiffness 1 year	23.1	21.8	25.0	23.7	0.5451
WOMAC function 1 year	21.4	21.4	22.7	22.5	0.6734
WOMAC total 1 year	20.1	19.6	21.6	20.9	0.6541
ROM 1 year (°)	110.1	12.2	109.5	14.7	0.338

*mFTA preop* preoperative mechanical femorotibial angle (varus > 180°, valgus < 180°), *WOMAC* Western Ontario and McMaster Universities osteoarthritis index, *ROM* range of motion, *MMV* medial midvastus approach, *MPP* medial parapatellar approach, *SD* standard deviation

In the MMV group the WOMAC total improved from 51.6 preoperatively to 20.1 at 1 year postoperatively. In the MPP group the WOMAC total improved from 51.2 preoperatively to 21.6 at 1 year postoperatively. At 1 year postoperatively there were no significant differences between groups in either WOMAC total or in the three WOMAC subscores (hypothesis 1, Table 1).

Preoperative ROM was 109.5° in the MMV group and 108° in the MPP group (*p* = 0.2819). Early postoperatively on days 4 and 10 after TKA there were also no differences between groups (*p* = 0.3049 and *p* = 0.3828, hypothesis 2, Table 1). Likewise, ROM was not significantly different between the groups 1 year after TKA (*p* = 0.3376, hypothesis 3, Table 1).

**Fig. 2:**
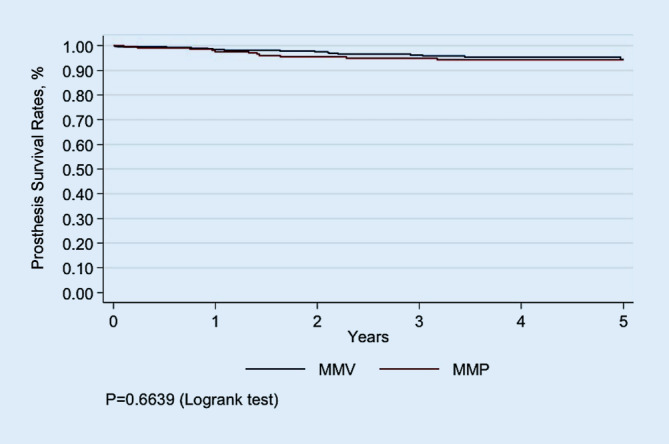
Graph showing prosthesis survival for the medial midvastus approach (*MMV*) group and the medial parapatellar approach (*MPP*) group as provided by the state arthroplasty registry

The 5‑year prosthesis survival did not differ between groups and showed 94.46% (95% CI 90.69–96.73%) in the MMV group and 94.33% (95% CI 89.96–96.83%) in the MPP group (*p* = 0.6639, hypothesis 4, Fig. 2).

## Discussion

It is regarded as the most important finding that the surgical approaches MMV and MPP did not significantly differ with respect to patient-reported knee score outcome (WOMAC), short-term postoperative ROM (day 4 and day 10), long-term postoperative ROM (1 year) or prosthesis survival.

For the purpose of comparing these findings with those of previous researchers a comprehensive literature analysis was performed. The search term “total AND knee AND (arthroplasty OR replacement) AND midvastus” produced 94 matches. After excluding irrelevant studies and those comparing approaches other than MMV and MPP there remained 15 original research articles [1, 2, 4–7, 10, 14, 16, 17, 19–21, 23, 25]. While three investigated solely surrogate parameters [7, 16, 21] (e.g. intraoperative tension in the lateral retinaculum), the remaining 12 studies (Table 2) provided clinical outcome, at least in part: knee scores, such as WOMAC and KSS, early and late postoperative ROM, VAS for pain, SLR ability, other rehabilitation milestones etc. (Table 2). At first glance it seems highly incongruent because six of those studies favored the MMV approach [1, 2, 4, 19, 23, 25] while the other six studies reported no such advantages of the MMV approach [5, 6, 10, 14, 17, 20]; however, when only considering very early postoperative follow-up intervals there seems to be some agreement amongst the authors that the MMV approach led to slightly better findings [1, 2, 19, 23, 25]. In detail, those studies tested parameters, such as quadriceps strength, time that SLR was possible and pain. In a synopsis of the previous literature and also taking into account the findings of the current study, MMV seems to have some advantages regarding rehabilitation in the early postoperative weeks, differences which disappear over time with most of them already after 2 months.

**Table 2:** Current knowledge on clinical outcome of total knee arthroplasty performed with either the medial midvastus (MMV) or the medial parapatellar (MPP) approach

Author	Year	*n*	Outcome parameters	FU periods	Results	Resumé
Aslam et al. [1]	2017	42 vs. 42	KSS, VAS, SLR, extensor lag, hospital stay, LRR rate, blood loss, patellar tracking	Day 1, 1 week, 1 month, 3 months, 6 months, 1 year	MMV: better KSS at 1 week and 1 month, earlier SLR, less LRR, shorter stay	Pro MMV
Bäthis et al. [2]	2005	25 vs. 25	VAS, Q‑strength, proprioception	3 weeks, 6 weeks	MMV: less pain, higher Q‑strength, no difference in ROM	Pro MMV
Dalury and Jiranek [4]	1999	24 vs. 24	Q‑strength, VAS, SLR, ROM at discharge, radiographic outcome	6 weeks, 12 weeks	MVV: higher Q‑strength at 6 and 12 weeks, less pain and earlier SLR	Pro MMV
Dalury et al. [5]	2008	20 vs. 20	Radiograph, electromyography, nerve conduction studies, ROM tests, and KSS, VAS, hospital stay, blood loss, radiographic outcome	6 weeks, 12 weeks	No difference	Equal
Engh et al. [6]	1997	61 vs. 57	LRR rate, patellar tracking, Q‑strength, ROM, SLR	6 weeks	No difference	Equal
Gelfer et al. [10]	2003	30 (both groups)	Patellar perfusion (bone scan), AKP, grind test, HSS	2, 3, 6 weeks	No difference	Equal
Keating et al. [14]	1999	100 vs. 100	LRR, ROM day 2, ROM at discharge, SLR, extensor lag	First weeks	No difference	Equal
Layher et al. [17]	2016	9 vs. 10	3D gait analysis (5 weeks, 6 months), KSS; WOMAC, SLR, STS, VAS, ROM at discharge, radiographic outcome	5 weeks, 6 months	MMV: worse ROM at discharge, better sagittal knee moment (5 weeks) and knee power (5 weeks, 6 months), better WOMAC 6 months	Equal
Maestro et al. [19]	2000	25 vs. 17	KSS, ROM, LRR rate, ROM, active knee extension	1, 6, 12 months	MMV: less LRR, better active knee extension day 5, better ROM at discharge	Pro MMV
Nutton et al. [20]	2014	12 vs. 16	Walking, stairs, SLR, stay, inpatient mobility milestones, knee kinematics, muscle strength, timed up and go, WOMAC, and daily step count, Q‑strength, SLR	6 weeks, 3 months, 6 months	No difference: time to walking, stairs, SLR, discharge, timed up and go, WOMAC, ROM, extensor strength	Equal
Shukla et al. [23]	2017	24 vs. 28	KSS; duration of hospital stay, blood loss	2 weeks, 6 weeks, 3 months, 6 months, 1 year	MVV: better KSS 2 weeks, 6 weeks, 3 months	Pro MMV
White et al. [25]	1999	109 vs. 109	Surgical time, LRR rate, and total blood loss. pain, ROM, SLR	Day 8, 6 weeks, 6 months	MVV: less LRR, less pain at day 8 and 6 weeks, better SLR at day 8	Pro MMV

*LRR* lateral retinacular release, *ROM* range of motion, *KSS* Knee Society score, *SLR* straight leg raise, *STS* sit to stand, *VAS* visual analogue scale (of pain), *HSS* Hospital for Special Surgery score, *AKP* anterior knee pain, *Q‑strength* quadriceps strength, *WOMAC* Western Ontario and McMaster Universities osteoarthritis index, *FU* follow-up

Regarding sample size most of the previous studies recruited around 50 patients for both groups together. Only 2 of the previous studies reported much higher sample sizes of around 200 for both groups together [14, 25]. The findings of the study at hand are highlighted by the fact that it is by far the largest study conducted so far with an overall sample size of more than 600. The current study is also the first investigation to link the issue of surgical approaches to prosthesis survival. The fact that both surgical approaches resulted in similar prosthesis survival suggests that the MMV approach obviously did not exert negative influences on TKA longevity.

The following limitations of the study are acknowledged. The outcome parameters were predominantly mid-term (1-year WOMAC, 1‑year ROM, 1–5-year survival). Only few parameters dealt with early postoperative function (day 4 ROM and day 10 ROM). This must be regarded as a limitation because potential early postoperative benefits of the MMV approach might therefore have been overlooked. Parameters, such as SLR, stair climbing, Q‑strength would have been of additional value. Also, it would have been beneficial to include knee score data, such as the WOMAC from 3 months postoperative. Other limitations are the facts that the patients were operated on by a large variety of surgeons and that no outcome parameters other than those mentioned above could be collected due to the retrospective nature of the study. The following facts are regarded as strengths of the study. It was the largest study conducted to date with an overall sample size of more than 600. Data quality is regarded as very robust as data originate from the state arthroplasty registry. Another advantage was that for the first time the question of the surgical approach in TKA was linked to prosthesis survival as reported from an arthroplasty registry.

The clinical relevance of the findings is regarded as high. Both surgical approaches may be used in daily practice during TKA. Using the MMV approach does not negatively affect prosthesis survival in the long run.

## Conclusion

On the basis of the findings, it is concluded that both surgical approaches in primary TKA, MMV and MPP, produce equivalent clinical results in terms of early postoperative ROM, late postoperative ROM and knee score outcome. The same prosthesis survival rates can be expected when using the MMV or the MPP approach in primary TKA. Consequently, the question whether to perform MMV or MPP depends on the surgeon’s preference.

## Abbreviations

IRB: Institutional review board
KSS: Knee Society score
LRR: Lateral retinacular release
mFTA: Mechanical femorotibial angle
MMV: Medial midvastus approach
MPP: Medial parapatellar approach
Q‑strength: Quadriceps muscle strength
ROM: Range of motion
SLR: Straight leg raise
TKA: Total knee arthroplasty
VAS: Visual analogue scale
WOMAC: Western Ontario and McMaster Universities osteoarthritis index
